# Effect of Psychological Care Combined with Traditional Chinese Medicine on Postoperative Psychological Stress Response in Patients with Advanced Cervical Cancer

**DOI:** 10.1155/2021/5612925

**Published:** 2021-09-28

**Authors:** Xue Hou, Huajuan Zhang, Yahong Nie, Yun Zhang, Jinhua Wang, Hua Xu

**Affiliations:** ^1^Department of Clinical Laboratory, Yantaishan Hospital, Yantai 264000, China; ^2^Hospital-Acquired Infection Control Department, Jinan Zhangqiu District Hospital of Traditional Chinese Medicine, Jinan 250200, China; ^3^Department of Clinical Laboratory, Affiliated Qingdao Central Hospital, Qingdao University, Qingdao 266042, China; ^4^Department of Paediatrics, Zhangqiu District People's Hospital, Jinan 250200, China; ^5^Department of Imaging, Zhangqiu District People's Hospital, Jinan 250200, China; ^6^Outpatient Operating Room, Affiliated Qingdao Central Hospital, Qingdao University, Qingdao 266042, China

## Abstract

**Objective:**

To study the effects of psychological care combined with traditional Chinese medicine treatment on the postoperative psychological stress response and the expression levels of serum C-reactive protein (CRP) and interferon-*γ* (IFN-*γ*) in patients with advanced cervical cancer.

**Method:**

232 postoperative advanced cervical cancer patients treated in our hospital from December 2015 to December 2018 were selected as study objects and divided into the control group and study group using the random number table method. The control group was given basic care combined with traditional Chinese medicine treatment, while the study group was given psychological care treatment on the basis of the control group to compare the treatment effect, psychological stress response, pain level, quality of life, and long-term efficacy of the two groups. The serum CRP and IFN-*γ* levels and their correlation with different psychological stress responses were compared between the two groups before and after treatment.

**Result:**

Comparing the clinical efficacy of the two groups, the total effective rate of the study group was higher than that of the control group. VAS scores in the study group were significantly lower than those in the control group 30 and 60 days after treatment. The SCL-90 scores of the study group after treatment were lower than those of the control group. After treatment, the differences between the two groups were statistically significant in the scores of emotional function, social function, and role function. The two-year cumulative survival rate in the study group (82.76%) was significantly increased compared to that in the control group (55.17%). The serum CRP and IFN-*γ* expression levels in the two groups were significantly decreased after treatment compared to those before treatment, and the serum CRP and IFN-*γ* expression levels in the study group were significantly decreased compared to those in the control group.

**Conclusion:**

Psychological care combined with traditional Chinese medicine in the treatment of advanced cervical cancer patients after surgery was effective in improving patients' psychological status, reducing their pain level, relieving postoperative negative emotions, increasing compliance, improving the quality of life, helping to prolong survival time, and controlling serum indexes back to normal, which was worth promoting in clinical practice.

## 1. Introduction

Cervical cancer is a serious common gynecologic malignant tumor disease located in the cervix, with high morbidity and mortality rates, and in recent years, the age of onset is gradually younger and the incidence is showing an increasing trend [1]. The cure rate of early cervical cancer is high and the prognosis is good. Some research data show that the earlier the clinical stage of cervical cancer, the higher the cure rate. For example, the five-year survival rate of stage la1 patients is as high as 97.5%. However, because the early onset of cervical cancer is more insidious, it is often at a locally advanced stage when diagnosed. Although modern medical technology is developing, the effect of surgery alone is poor and the survival rate is low, which seriously endangers the life of patients [2,3].

At present, one of the key approaches of treating cervical cancer is still surgery, supplemented by radiotherapy, and one of the important reasons affecting the prognosis is the late clinical stage [4]. Patients with intermediate and advanced cervical cancer often show symptoms such as cold and weakness, pallor, nausea and vomiting, and fat tongue during chemotherapy; cervical nodules; vaginal bleeding; and lower abdominal pain [5]. Surgical trauma, economic stress, and postoperative and disease pain may lead to severe psychological stress reactions in patients, which may lead to disruption of their physiological functions. Moreover, patients are prone to insomnia, fatigue, anorexia, and so on, which seriously degrades patients' quality of life and affects the clinical treatment outcome [6]. Therefore, postoperative psychological care is crucial to improve the level of psychological stress in patients. In recent years, the clinical application of traditional Chinese medicine has become more and more widespread, and traditional Chinese medicine has the efficacy of tonifying the spleen and kidney, tonifying qi and blood, activating blood circulation, removing blood stasis, and so forth and has less adverse effects and high safety profile, which is increasingly favored by doctors and patients [7].

In this study, 232 postoperative patients with advanced cervical cancer admitted to our hospital were divided into a study group and a control group. They were given basic care, traditional Chinese medicine treatment combined with psychological care treatment, respectively, to explore the effects of psychological care combined with traditional Chinese medicine treatment on the psychological stress response and serum indexes of patients with advanced postoperative cervical cancer.

## 2. Materials and Methods

### 2.1. General Data

A total of 232 postoperative patients with advanced cervical cancer treated at the Yantaishan Hospital, Yantai, Shandong, China, from December 2015 to December 2018 were included in this study. They were pathologically confirmed and randomly divided into a study group and a control group, with 116 cases in each group, using the random number table method. This study was approved by the ethics committee of the Yantaishan Hospital, Yantai, Shandong, China.

Inclusion criteria: all Chinese medicine diagnoses met the diagnostic criteria in the Guidelines for Clinical Research on New Chinese Medicines [8]; all Western medicine diagnoses met the diagnostic criteria in the Guidelines for the Diagnosis and Treatment of Common Gynecologic Malignancies [9]; the expected survival period was ≥6 months; and all patients were informed about the study purpose and signed an informed consent prior participation.

Exclusion criteria: pregnant and lactating women; patients with severe cardiac, hepatic, and renal dysfunction; patients with immune deficiency; patients with other tumor diseases; patients with impaired consciousness and uncooperative treatment; patients with intolerance or allergy to the study drugs and drug components; patients who had received antitumor treatment such as radiotherapy or chemotherapy in the 1 month before treatment.

There was no statistically significant difference between the general data of the two groups (*P* > 0.05, Table 1).

### 2.2. Treatment Methods

#### 2.2.1. Chinese Medicine Treatment

Patients in both groups were given basic care combined with Chinese herbal medicine treatment. The Chinese herbal formula was as follows: galanga galangal fruit 10 g, white mulberry root-bark 10 g, heartleaf houttuynia herb 30 g, eucommia bark 20 g, poria 10 g, milkvetch root 30 g, largehead atractylodes rhizome 15 g, coix seed 20 g, garden burnet root 25 g, willowleaf rhizome 5 g, Chinese angelica 5 g, cassia bark 30 g, lightyellow sophora root 15 g, peony root 10 g, milkwort root 15 g, and atractylodes rhizome 25 g. Decoct the above herbs in water to 300 mL. Take one dose a day, divided into morning and evening. Fifteen days was a course of treatment, followed by four courses of treatment.

### 2.3. Psychological Care Treatment

Communicate properly in detail with patients and their families with no language barrier, make a comprehensive evaluation of the patient's psychological condition, understand the patient's condition and the patient's needs, encourage them to talk about their psychological concerns, and instruct their families to participate in the deescalation work to increase the patient's sense of security. Patients were relieved of adverse psychological emotions through music therapy, conversation method, emotional catharsis, and so on. Remove the misconceptions of patients and their families through health education and enhance the level of patients' knowledge about cervical cancer and their awareness about surgery. Tell patients about successful treatment cases at the right time to further enhance patients' confidence in treatment. Nursing staff should care for patients' inner feelings according to their personality characteristics, inform them about the operation in time and answer their questions patiently. In addition, patients can also be encouraged to participate in social activities appropriately according to their recovery. Special attention was given to those with more severe psychological stress. The patient's postoperative pain level was assessed, and the patient was helped to relieve pain and maintain a good state of mind through deep breathing, distraction, and analgesic drugs.

### 2.4. Observation Indicator

Efficacy evaluation: the evaluation was made by imaging the changes in tumor size of patients, and the tumor volume was calculated as length∗width∗radius. The efficacy evaluation was performed according to the WHO solid tumor efficacy criteria [10]. The complete disappearance of all visible tumor lesions for more than 4 weeks was considered CR; a decrease of more than 50% in the product of two tumor diameters and no new lesions appearing was considered PR; a decrease of less than 50% in the product of two tumor diameters and an increase of no more than 25% was considered SD; and an increase of more than 25% in the product of two tumor diameters or the appearance of new lesions was considered PD. Total effective rate = (CR + PR)/total number of cases. A visual analogue scale [11] (VAS) was used to assess the pain level of cervical cancer patients at 1 d, 7 d, 30 d, and 60 d postoperatively, and patients were asked to mark the position representing their pain level in a 10 cm walking scale (with the scale face back to the patient), with a score of 0 being no pain and 10 being the most severe pain; the higher the score, the more severe the pain level.

The Symptom Checklist 90 (SCL-90) [12] was used to evaluate changes in patients' psychological stress levels. The scale has 10 factors, that is, reflecting the patient's psychological symptoms from somatization, obsessive-compulsive symptoms, interpersonal sensitivity, depression, anxiety, hostility, phobia, paranoia, psychoticism, and others (e.g., sleep and eating). There are 90 entries, each factor has 9 entries, each entry has 1 to 5 points, and each factor has 9 to 45 points, with 16 points as the standard cut-off value; the higher the score, the more severe the level of psychological stress. Quality of life scores were mainly measured using the SF-36 scale [13], which measured five dimensions of physical, emotional, social, cognitive, and role functioning, with higher scores indicating better quality of life.

The expression levels of serum CRP and IFN-*γ* in the two groups of patients were compared before and after treatment. The long-term efficacy of the two groups was compared, and the two-year cumulative survival rate was counted and calculated at the end of treatment with a two-year follow-up.

### 2.5. Statistical Analysis

Statistical analysis was performed using SPSS 23.0 statistical software, and the measurement data were compared using a *t*-test, expressed as mean ± standard deviation. The count data were compared using the chi-square test, expressed as a percentage, with *P* < 0.05 indicating a significant difference in statistical tests.

## 3. Results

### 3.1. Comparison of Clinical Efficacy between Two Groups of Patients

After treatment, the total effective rate of the study group (87.07%) was significantly increased compared with that of the control group (64.66%, Table 2).

### 3.2. Comparison of VAS Scores after Treatment between the Two Groups

**Figure 1:**
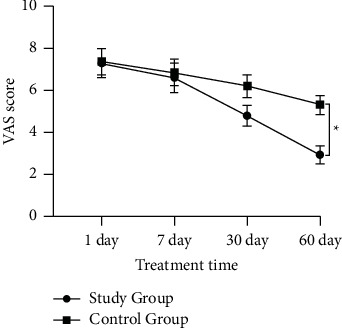
Comparison of VAS scores of the patients after treatment between the control group (*n* = 116) and the study group (*n* = 116). ^*∗*^*P* < 0.05.

The difference in VAS scores between the two groups was not statistically significant at 1 day of treatment (*t* = 0.415, *P* = 0.852) and 7 days of treatment (*t* = 0.423, *P* = 0.556); at 30^th^ day of treatment, the VAS score was (4.80 ± 0.323) in the study group and (6.200 ± 0.317) in the control group, and the difference was statistically significant (*t* = 4.341, *P* = 0.002); on treatment day 60, the VAS score was (2.94 ± 0.278) in the study group and (5.32 ± 0.271) in the control group, and the difference was statistically significant (*t* = 8.577, *P* ≤ 0.001) (Figure 1).

### 3.3. Comparison of SCL-90 Scores between the Two Groups before and after Treatment

Before treatment, there was no statistically significant difference between the SCL-90 scores of the two groups (*P* > 0.05). After treatment, the SCL-90 scores of the two groups decreased (*P* < 0.05), and the decrease was more significant in the study group (*P* < 0.05) (Table 3).

### 3.4. Comparison of Serum CRP and IFN-*γ* Expression Levels before and after Treatment between the Two Groups of Patients

**Figure 2:**
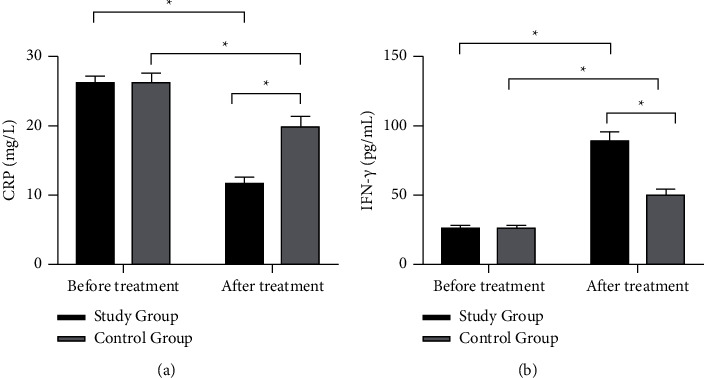
Comparison of serum CRP and IFN-*γ* expression levels before and after treatment between the control group (*n* = 116) and the study group (*n* = 116). (a) The comparison of serum CRP expression levels before and after treatment between the control group (*n* = 116) and the study group (*n* = 116). (b) The comparison of serum IFN-*γ* expression levels before and after treatment between the control group (*n* = 116) and the study group (*n* = 116). ^*∗*^*P* < 0.05.

Before treatment, there was no statistically significant difference in the expression levels of serum CRP and IFN-*γ* between the two groups (*P* > 0.05); after treatment, serum CRP levels were downregulated in both groups compared with those before treatment (*P* < 0.05), and the downregulation was more significant in the study group. IFN-*γ* levels were upregulated after treatment in both groups compared with pretreatment (*P* < 0.05), and the upregulation was more significant in the study group (*P* < 0.05) (Figure 2).

### 3.5. Comparison of Quality of Life Scores between the Two Groups

After treatment, the differences in emotional, social, and role function scores between the two groups were statistically significant (*P* < 0.05), while the differences in physical and cognitive function scores were not statistically significant (*P* > 0.05) (Table 4).

### 3.6. Comparison of Long-Term Efficacy between the Two Groups of Patients

**Figure 3:**
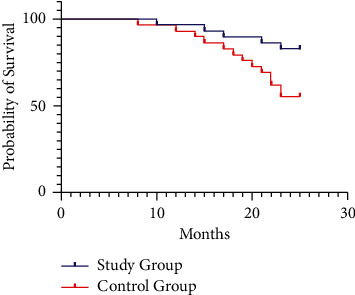
Comparison of long-term outcomes between the control group (*n* = 116) and the study group (*n* = 116).

The two-year cumulative survival rate in the study group (82.76%) was significantly increased compared to that in the control group (55.17%) (*P* < 0.05, Figure 3).

## 4. Discussion

Cervical cancer is a gynecologic malignancy with high morbidity and mortality, with many new cases every year. In recent years, some studies have reported that the incidence of the disease is gradually increasing in young age, and at an advanced stage, the treatment and prognosis are poor and the death rate is high [14]. With the progress of socioeconomic and medical science, more and more clinicians gradually realize that psychological factors have an important influence on the occurrence of tumor, disease progression, treatment effect, and prognosis and have a high clinical value [15]. At present, psychological intervention for tumor patients has become a hot topic in clinical research. Li et al. [16] found that due to the diagnosis of cervical cancer and the impact of surgical treatment, patients usually face tremendous physiological-psychological and social stresses after surgery. Coupled with postoperative physical pain and lack of understanding and uncertainty about the disease, patients will have serious psychological stress reactions, such as anxiety, depression, and insomnia. Psychological interventions are urgently needed to make them feel warm, comforted, and happy in the process of fighting cancer; safeguard patients' psychological health; and enhance their treatment confidence, which is conducive to enhancing treatment compliance and promoting recovery. Shi et al. [17] showed that psychological interventions could also reduce the levels of inflammatory factors, such as CRP, in patients with cervical cancer. As an inflammatory factor, CRP reflects the inflammatory condition and the degree of damage in the body. In normal human serum, the level of CRP is low, and when its level is abnormally elevated, it indicates that the patient's condition is worsening [18]. The results of this study showed that after treatment, the serum CRP levels of patients significantly decreased, indicating that psychological care combined with traditional Chinese medicine in the treatment of postoperative advanced cervical cancer patients can significantly reduce the level of the inflammatory factor CRP and enhance the immunity of the body, which is consistent with the results of our study [17]. IFN-*γ* is a potent antiviral and antitumor interferon and mainly regulates the immune system, which can induce T cells to enhance their ability to recognize tumor antigens and ultimately, the body's ability to monitor tumors [19]. Studies have shown [20] that serum IFN⁃*γ* levels are significantly downregulated with the aggravation of cervical lesions. The present study was consistent with these findings.

In the Chinese medicine health system, cervical cancer belongs to leukorrheal diseases and the dialectical treatment should focus on clearing heat and removing toxins [21]. The formula selected for this study consisted of 16 herbs. Chinese angelica has good effect on promoting blood circulation for regulating menstruation and relieving pain, which can effectively relieve acute pain; peony root has the effect of dispersing blood stasis, clearing heat, cooling blood, and relieving pain; lightyellow sophora root has the effect of invigorating spleen-stomach and replenishing qi; coix seed has the effect of detoxification and discharging pus; galanga galangal fruit has the effect of invigorating spleen and resolving food stagnation, eliminating dampness and dispelling cold; white mulberry root-bark has the effect of purging lung for relieving asthma, diuresis, and detumescence; heartleaf houttuynia herb has the effect of clearing heat and removing toxicity, anti-inflammatory and antiviral; eucommia bark has the effect of nourishing liver and invigorating the kidney; poria can induce diuresis for removing edema, reinforce spleen, relieve diarrhea, reinforce heart and spleen, and nourish the heart to tranquilize; milkvetch root has the effect of reinforcing qi strengthening exterior defence and inducing diuresis for removing edema; largehead atractylodes rhizome has the effect of invigorating vital energy, invigorating spleen, and eliminating dampness and diuresis; garden burnet root has the effect of cooling blood for hemostasis, detoxication, and astringing sores; willowleaf rhizome has the effect of purging lung and descending qi; cassia bark and atractylodes rhizome have the effect of warmly invigorating kidney yang, dispelling cold for relieving pain, warming meridians, and activating blood; and milkwort root has the effect of tranquillization and expelling phlegm [22–26]. The combination of these drugs exerts the effects of clearing heat and removing toxicity, eliminating dampness, and promoting blood circulation.

The results of this study showed that the total effective rate of the study group was significantly higher than that in the control group (*P* = 0.001). The pain level of patients in the study group was significantly lower than that in the control group after 30 days and 60 days of treatment (*P* < 0.05). The level of psychological stress in the study group after care was significantly lower than that in the control group, indicating that psychological care combined with herbal treatment was effective in the treatment of postoperative advanced cervical cancer patients and could effectively improve the degree of postoperative pain and the adverse psychological reactions of patients. Breitbart et al. [27] showed that a positive and healthy state of mind was conducive to disease recovery. Cunningham [28] showed that psychological interventions could reduce postoperative pain and improve psychological status by reducing pain and oxidative stress factors in patients with cervical cancer. The results of this study showed that the serum IFN-*γ* level of patients increased significantly after treatment and the IFN-*γ* level was negatively correlated with the degree of psychological stress of patients, indicating that psychological care combined with traditional Chinese medicine treatment could effectively increase the serum IFN-*γ* level of patients, improve the antiviral ability of the body, and enhance the immunity of the body. After treatment, the patients in the study group had a significantly higher emotional function, social function, and role function scores than the control group (*P* < 0.05). The two-year cumulative survival rate was significantly higher than that of the control group (*P* < 0.05). Taken together, psychological care combined with traditional Chinese medicine treatment was more effective for postoperative advanced cervical cancer patients, which can significantly improve patients' quality of life and prolong their survival time.

## 5. Conclusion

In conclusion, psychological care combined with traditional Chinese medicine treatment could significantly improve the psychological stress response of patients with advanced cervical cancer, reduce the degree of the physical pain of patients, improve the quality of patients' life, and prolong the life of patients.

## Figures and Tables

**Table 1:** Comparison of general information between the two groups of patients.

Indicator	Control group	Study group	*X* ^2^	*P* value
Case	116	116		
Age (years)			0.070	0.791
>43	67	65		
≤43	49	51		
Course of the disease (years)			0.322	0.570
<3	78	82		
3-4	38	34		
Educational level			0.078	0.780
High school and below	64	60		
Specialized degree and above	52	56		
BMI			0.088	0.957
<18.5	17	16		
18.5–24.9	84	86		
≥25	15	14		
Clinical stage			0.254	0.881
IIIa	47	48		
IIIb	45	47		
IVa	24	21		
Pathological type			0.248	0.618
Squamous cell carcinoma	92	95		
Adenocarcinoma	24	21		

**Table 2:** Comparison of clinical efficacy between two groups of patients (*n* (%)).

Group	*n*	CR	PR	SD	PD	Total effective rate
Control group	116	24	77	9	6	101 (87.07)
Study group	116	13	62	28	13	75 (64.66)
*X* ^2^						17.225
*P* value						0.001

**Table 3:** Comparison of SCL-90 scores before and after treatment in the two groups (‾*x* ± *s*).

Group		*n*	Somatization	Interpersonal sensitivity	Anxiety	Phobia	Psychoticism
Study group	Before treatment	116	36.72 ± 3.45	39.16 ± 4.23	38.34 ± 4.71	35.96 ± 3.37	36.43 ± 4.52
	After treatment	116	12.56 ± 2.13	11.48 ± 2.76	12.14 ± 3.48	10.87 ± 2.26	10.93 ± 2.17
*t*			11.263	13.861	23.654	4.782	7.665
*P*			<0.05	<0.05	<0.05	<0.05	<0.05
Control group	Before treatment	116	35.63 ± 3.36	39.87 ± 3.94	37.81 ± 5.27	35.57 ± 3.44	35.67 ± 4.31
	After treatment	116	21.34 ± 2.71	22.51 ± 2.32	21.62 ± 4.47	19.26 ± 2.31	20.42 ± 3.32
*t*			7.036	8.541	11.230	3.117	6.548
*P*			<0.05	<0.05	<0.05	<0.05	<0.05
		*n*	Obsessive-compulsive symptoms	Depression	Hostility	Paranoia	Others
Study group	Before treatment	116	35.76 ± 4.41	38.97 ± 4.68	36.62 ± 5.53	37.73 ± 4.54	38.24 ± 4.21
	After treatment	116	11.05 ± 2.61	11.27 ± 2.46	10.92 ± 2.84	11.21 ± 2.53	12.57 ± 2.72
*t*			15.423	8.662	11.346	12.951	7.652
*P*			<0.05	<0.05	<0.05	<0.05	<0.05
Control group	Before treatment	116	35.78 ± 3.83	37.95 ± 5.19	36.15 ± 5.26	36.75 ± 5.66	38.87 ± 4.53
	After treatment	116	21.53 ± 2.44	21.66 ± 3.23	20.41 ± 3.27	21.58 ± 3.68	20.54 ± 3.17
*t*			8.932	6.231	7.145	10.213	6.430
*P*			<0.05	<0.05	<0.05	<0.05	<0.05

**Table 4:** Comparison of quality of life scores between the two groups (‾*x* ± *s*).

Group	*n*	Somatic function	Emotional function	Social function	Cognitive function	Character function
Study group	116	91.24 ± 5.67	88.36 ± 5.24	89.64 ± 6.53	90.77 ± 6.15	87.23 ± 7.96
Control group	116	86.14 ± 4.78	72.45 ± 4.57	74.23 ± 5.74	88.66 ± 5.36	71.41 ± 6.28
*t*		0.352	7.452	6.781	0.730	4.512
*P*		>0.05	<0.05	<0.05	>0.05	<0.05

## Data Availability

The data used to support the findings of this study are available from the corresponding author upon request.
